# Structure Prior Effects in Bayesian Approaches of Quantitative Susceptibility Mapping

**DOI:** 10.1155/2016/2738231

**Published:** 2016-12-20

**Authors:** Shuai Wang, Weiwei Chen, Chunmei Wang, Tian Liu, Yi Wang, Chu Pan, Ketao Mu, Ce Zhu, Xiang Zhang, Jian Cheng

**Affiliations:** ^1^School of Electronic Engineering, University of Electronic Science and Technology of China, Chengdu, Sichuan, China; ^2^Center for Robotics, University of Electronic Science and Technology of China, Chengdu, Sichuan, China; ^3^Department of Radiology, Tongji Hospital, Tongji Medical College, Huazhong University of Science and Technology, Wuhan, Hubei, China; ^4^School of Computer Science and Engineering, Wuhan Institute of Technology, Wuhan, Hubei, China; ^5^Medimagemetric LLC, New York, NY, USA; ^6^Department of Biomedical Engineering, Cornell University, Ithaca, NY, USA; ^7^Department of Radiology, Weill Cornell Medical College, Cornell University, New York, NY, USA

## Abstract

Quantitative susceptibility mapping (QSM) has shown its potential for anatomical and functional MRI, as it can quantify, for* in vivo* tissues, magnetic biomarkers and contrast agents which have differential susceptibilities to the surroundings substances. For reconstructing the QSM with a single orientation, various methods have been proposed to identify a unique solution for the susceptibility map. Bayesian QSM approach is the major type which uses various regularization terms, such as a piece-wise constant, a smooth, a sparse, or a morphological prior. Six QSM algorithms with or without structure prior are systematically discussed to address the structure prior effects. The methods are evaluated using simulations, phantom experiments with the given susceptibility, and human brain data. The accuracy and image quality of QSM were increased when using structure prior in the simulation and phantom compared to same regularization term without it, respectively. The image quality of QSM method using the structure prior is better comparing, respectively, to the method without it by either sharpening the image or reducing streaking artifacts* in vivo*. The structure priors improve the performance of the various QSMs using regularized minimization including L1, L2, and TV norm.

## 1. Introduction

Quantitative susceptibility mapping (QSM) generates tissue magnetic susceptibility property image from susceptibility-sensitive MRI data [1–4]. QSM can reduce blooming artifacts in susceptibility weighted imaging [5], is clinically useful for quantifying magnetic biomarkers that have susceptibilities different from their surroundings [6–9], and promises to probe oxygen metabolism [10–12] and inflammation [13]. The basic QSM physics includes estimating the local magnetic field **δ**_*b*_(**r**) (measured main magnetic field **B**_0_) from the MRI signal phase [14, 15] and modeling the field as from dipole sources in tissue*:* **δ**_*b*_ = **d** ⊗ **χ** + **n** in image space (referred to as **r**-space)* or *Δ_*b*_ = **D** **X** + **N**  in **k**-space, where **d** is a unit dipole in **r**-space (referred as dipole kernel), **χ** is the tissue susceptibility distribution in **r**-space, **n** is the observed noise in **r**-space, and Δ_*b*_, **X** ****, **D**, and **N** are corresponding to **δ**_*b*_, **d**, **χ**, and **n** in **k**-space, respectively [16–18]. The fundamental QSM algorithm is to solve the inverse problem from the field **δ**_*b*_ to the susceptibility source **χ**.

Because the dipole kernel has a pair of opposing zero cone surfaces at the magic angle (54.7°) with respect to the **B**_0_ direction, the inversion from field to susceptibility is fundamentally ill-posed, and QSM requires additional information to select a unique susceptibility map from many possibilities [19]. For clinical data acquired using a single orientation, various regularization methods have been proposed for QSM [17]. The Bayesian formulation provides the main QSM approach to identify a susceptibility distribution of minimal streaking artifacts [20–22].

Bayesian QSM requires constructing a data fidelity term according to data noise property and a prior information term. Previously, we examine the importance of noise whitening in constructing the data fidelity term [17]. Here, we examine the importance of including structure information in the prior term. Specifically, we analyze the effects of the choice of prior in QSM based on three published methods in literature and several new algorithms with different regularization terms. All QSM methods are evaluated using simulations and phantom experiments where the susceptibility is known. Further, clinical applicability of QSM was evaluated with human brain data to investigate image quality.

## 2. Material and Methods 

In Bayesian approach, the regularization prior is expressed as a cost function **R** that favors a solution of the desired property, and the degree to which it is favored is typically characterized by a regularization parameter *α*. The maximum a posterior solution [17, 23] is

(1) $$\begin{array}{c} \chi =\underset{\chi }{\arg\min}\left[\;E+\alpha R\;\right], \end{array}$$

where **E** constitutes the data fidelity term. Since Gaussian noise in the complex MR signal domain should be accounted for in the data fidelity term and with proper noise weighting, noise effects in QSM can be reduced using Bayesian methods [17]. In the following section, we use **E** = ‖**w** **z**‖_2_^2^, with **z** = **d** ⊗ **χ** − **δ**_*b*_ and **w**  as noise weighting.

### 2.1. Priors Used in Various Algorithms

The following examples of regularization terms have been explored [17]:

(i) The gradient (*G*) L2 norm (GL2)
  (2) $$\begin{array}{c} R={{\left\|G\chi \right\|}_{2}}^{2}={{\left\|\frac{\partial \chi }{\partial x}\right\|}_{2}}^{2}+{{\left\|\frac{\partial \chi }{\partial y}\right\|}_{2}}^{2}+{{\left\|\frac{\partial \chi }{\partial z}\right\|}_{2}}^{2} \end{array}$$
(ii) The gradient L1 norm (GL1)
  (3) $$\begin{array}{c} R={\left\|\;G\chi \;\right\|}_{1}={\left\|\;\frac{\partial \chi }{\partial x}\;\right\|}_{1}+{\left\|\;\frac{\partial \chi }{\partial y}\;\right\|}_{1}+{\left\|\;\frac{\partial \chi }{\partial z}\;\right\|}_{1} \end{array}$$
(iii) The total variation norm (TV)
  (4) $$\begin{array}{c} R=TV\left(\;\chi \;\right)=\underset{r}{\sum }\sqrt{{\left|\frac{\partial \chi }{\partial x}\left(r\right)\right|}^{2}+{\left|\frac{\partial \chi }{\partial y}\left(r\right)\right|}^{2}+{\left|\frac{\partial \chi }{\partial z}\left(r\right)\right|}^{2}} \end{array}$$
(iv) A wavelet domain such as a Daubechies wavelet (Φ) L1 norm
  (5) $$\begin{array}{c} R={\left\|\;\Phi \chi \;\right\|}_{1} \end{array}$$
(v) A combination of two sparsity terms such as total variation and wavelet [24]
  (6) $$\begin{array}{c} \alpha R⟶\alpha {\left\|\;\Phi \chi \;\right\|}_{1}+\beta TV\left(\;\chi \;\right) \end{array}$$
(vi) An L1 norm with structural consistency term **m** (MEDI) [25–28]
  (7) $$\begin{array}{c} R={\left\|\;mG\chi \;\right\|}_{1} \end{array}$$

We evaluated the quantification accuracy and image quality of various QSM algorithms using numerical and experimental phantom data and image quality in clinical applications with* in vivo* patient data.

### 2.2. QSM Algorithms in This Paper

Six representative algorithms are summarized in Table 1 and in pseudocodes in the Appendix for reference: MGL2 (GL2 with structural consistency prior **m**), MTV (TV with **m**), and MEDI (GL1 with **m**). The equations of the GL2 and MGL2 methods were solved using the conjugate gradient method (CG). The MEDI, TV, MTV, and GL1 methods were solved using a lagged diffusivity fixed point method (LDFP). In methods involving a structure prior, **m** was estimated by setting the gradients of magnitude image greater than a certain threshold to 0 and to 1 otherwise [28]. The threshold was adjusted iteratively such that approximately 30% of the voxels within the interested brain region were 0, that is, considered to have gradients. This threshold was determined from a previous theoretical study where the threshold was varied and a global minimum was empirically found at 30% [19].

We applied a one-dimensional temporal phase unwrapping and a linear least squares fitting to estimate the rate of phase evolution to get field map. The noise weighting  **w** was estimated as the SNR along with the field map estimation [17]. The PDF method [14] was used for removing the background field. The local field outside the brain parenchyma was cropped by a mask, which was manually segmented in the numerical and phantom experiments and was obtained using a Robust Brain Extraction (ROBEX) tool for* in vivo* brain data [29]. Voxels in the background region or within 3 mm to the background region were set to zero.

### 2.3. Numerical Simulation

**Figure 1:**
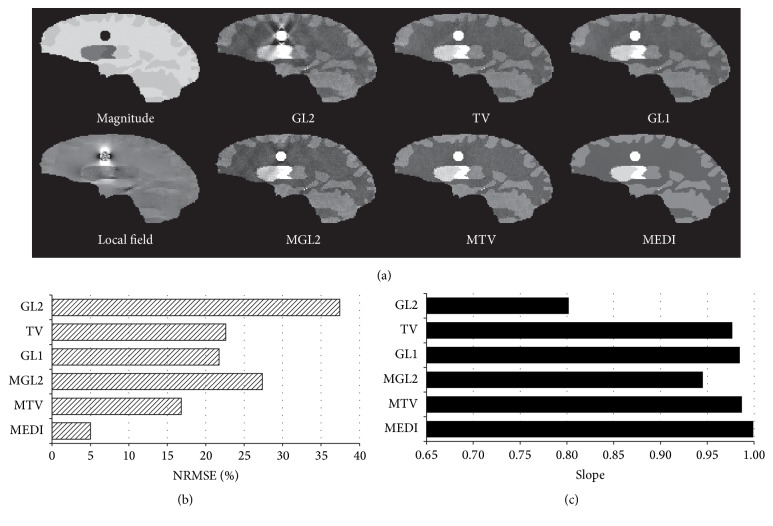
Regularization effects on QSM reconstructions demonstrated in a noisy Zubal lesion phantom: (a) T2^*∗*^ magnitude and local field, without (top) or with structure prior  **m** (bottom). The structural consistency reduced artifacts in susceptibility reconstructions for GL2, TV, and MEDI. This is supported with the quantitative measurements of (b) the relative errors and (c) regression slopes (all with *R* ^2^ > 0.98). The image quality of MEDI is also superior to MGL2 and MTV.

A 256 × 256 × 128 Zubal-type [30] numerical susceptibility phantom (Figure 1) was built containing a spherical lesion of 5-pixel radius to simulate a low SNR region within the T2^*∗*^ magnitude image of the brain parenchyma. The simulated susceptibility values were −0.05, 0.07, 0.09, 0.09, 0.19, 0.30, 0.90, and 0.00 ppm for white matter, thalamus, caudate nucleus, putamen, globus pallidus, veins, lesion, and other parenchyma, respectively. Complex Gaussian zero-mean noise with standard deviation ranging from 0.01 to 0.05 was added to the simulated complex MRI signal.

### 2.4. Phantom Experiments

A Gd phantom was constructed consisting of six spherical balloons filled with solutions of various concentrations of Gd-DTPA (Magnevist; Berlex Laboratories, Wayne, NJ) and immersed in water within a cylindrical container with dimensions of 12.5 cm diameter and 30 cm height. The Gd concentrations ranged from 0.5% to 3.0% (using a 0.5% increment) with susceptibilities ranging from 0.81 to 4.89 ppm, corresponding to the Gd concentration in aortic arch measured in the first pass of a dynamic contrast enhanced MRI [18]. This phantom was scanned on a 1.5T MRI scanner (HDx, GE Healthcare, Waukesha, WI) with a body coil. A T2^*∗*^ weighted multiecho gradient echo sequence was performed with the following parameters: FA = 30°; TR = 30 ms; number of TEs = 3; first TE = 3.05 ms; uniform TE spacing (ΔTE) = 1.0 ms; BW = ±31.2 kHz; FOV = 12.8 cm; and acquired resolution = 2 × 2 × 2 mm^3^.

### 2.5. * In Vivo* Brain Imaging

The human study was approved by our Institutional Review Board. Thirty-six patients who underwent brain MRI examination from January to September in 2015 were retrospectively included in this study including 18 consecutive cases without hemorrhagic lesions (group 1) and 18 consecutive cases with hemorrhages (group 2). All MR examinations were performed on a 3.0T MR system (Signa HDxt, GE, USA), using an 8 channel head coil. A 3D T2^*∗*^ weighted multiecho gradient echo sequence was used with the following parameters: FA = 20°; BW = ±41.67 kHz; field of view (FOV) = 24 cm; TR = 57 ms; number of echoes = 8; first TE = 5.7 ms; uniform TE spacing (ΔTE) = 6.7 ms; and acquired resolution 0.57 × 0.75 × 2  (*n* = 34) and 0.5 × 0.7 × 0.7  (*n* = 2) mm^3^.

### 2.6. Implementation Details for Algorithms

The regularization parameter (*α*) was searched from 10^−5^ to 10^1^(13 logarithmically spaced steps) for all QSM methods. The best parameter of every method for noisy numerical simulation and phantom was chosen according to the least error with respect to the true susceptibility [17, 31] and to the prepared susceptibility in each balloon. Because the true susceptibility is not available in the real human brain, the best parameter of each method is chosen according to the balance of artifacts and contrast among brain components in one representative case by the 13-year experienced neuroradiologist (W. C.) with inspecting all the varying parameter's results reconstructed by the method. And all real cases use the chosen parameters.

### 2.7. Data Analysis

The normalized root mean square error (NRMSE) (normalized by root mean square of true susceptibility) of the whole volume and the linear regression of the measured versus known susceptibility was calculated for every method. This NRMSE is used to assess the accuracy of the numerical phantom reconstruction. In the Gd phantom, a linear regression between the measured and prepared susceptibilities of the various balloons was performed for each of the methods.

To assess difference between two QSM algorithms in numerical and experimental phantoms, the values of susceptibilities are measured and compared to their known values. Their differences between two methods were assessed using paired *t*-test based on comparing linear regressions, and the method with the smaller error was reported as improvement when *p* < 0.05.

To assess the quality of patient images reconstructed by different QSM methods, three neuroradiologists (W. C., C. P., and K. M.) reviewed images simultaneously in a random order blinded to the reconstruction methods. Overall image quality was scored with a 5-point scale based on radiological impression of smoothness and artifacts, where 5 is the highest quality and 1 is the lowest quality [17]. The radiologist (W. C.) assessed the image quality again 6 months later to assess intraobserver variability. Interobserver and intraobserver variabilities of image quality scores were assessed by using the intraclass correlation coefficient [32]. The following criteria for clinically relevant agreement were used to assess the calculated intraclass correlation coefficient: less than 0.40 was considered poor; 0.40–0.59, fair; 0.60–0.74, good; and greater than 0.74, excellent [33]. The significance of image score differences between reconstructed susceptibility maps assessed by the Wilcoxon rank sum test. Statistically significant with the higher image score was reported as an improvement when *p* < 0.05.

## 3. Results

### 3.1. Numerical Simulation

The structural prior **m** markedly reduced streaking artifacts originating from the simulated lesion for GL2 (*p* = 9.22 × 10^−7^), TV (*p* = 0.02), and GL1 (*p* = 0.04) as seen in Figure 1(a), which was also reflected in the reduced NRMSE (Figure 1(b)) and in the increased regression slopes more closely approaching 1 as seen in Figure 1(c). The QSM reconstruction accuracy was improved from MGL2 to MTV (*p* = 3.49 × 10^−3^) and from MTV to MEDI (*p* = 0.03).

### 3.2. Phantom Experiments

The slopes of the regression analysis for GL2, MGL2, TV, MTV, GL1, and MEDI were 0.83, 0.92, 0.89, 0.94, 0.89, and 0.96, respectively. With the addition of the structure prior **m** the reconstructions showed increased regression slopes (*p* = 0.03, 0.02, 0.02 for GL2, TV, and GL1, resp.) approaching 1. The QSM reconstruction accuracy was improved from MGL2 to MTV (*p* = 0.03) and from MTV to MEDI (*p* = 1.63 × 10^−3^).

### 3.3. *In Vivo* Brain Imaging

**Figure 2 fig2:**
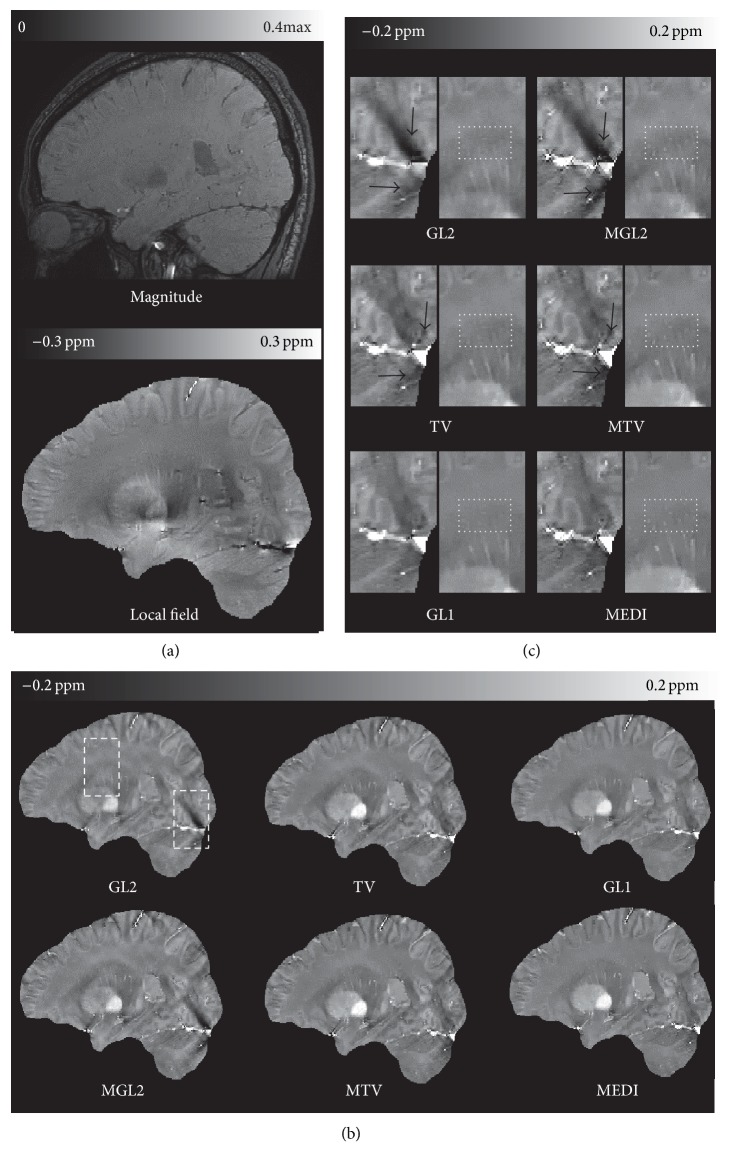
Structure regularization effects on a healthy subject in group 1. (a) magnitude, local field, and (b) reconstructed QSM images without (left) or with structure prior (right) in a sagittal section. The dashed boxes in (b) are zoomed in on (c). The structure consistency **m** reduces overall streaking artifacts comparing QSM methods with and without it. The streaking artifacts were seen originating from vessels (black arrows) in GL2 and MGL2 in (c). This artifact was reduced to some extent in TV and MTV and even further in GL1 and MEDI. The appearance of small veins (dot boxes in (c)) is also improved in the method with **m** compared to the method without it, respectively. The averaging image quality scores of this subject were 1, 2, 2.7, 3.3, 3.7, and 5 for GL2, MGL2, TV, MTV, GL1, and MEDI, respectively.

The average overall image quality scores (mean ± standard deviation) of QSMs are shown in Table 1 for every method, respectively. The spatial prior either sharpened the image or reduced streaking artifacts (Figure 2 black arrows). The overall image quality scores were higher when using structural consistency (**m**) prior compared to the same method without a structural consistency prior observed in the reconstructions of group 1. Compared to the method without **m**, the artifact from vessels (black arrow in Figure 2) is reduced and the appearance of small veins (dot boxes in Figure 2) is improved in the method with **m**, respectively. The difference is statistically significant (*p* = 6.37 × 10^−3^, 9.38 × 10^−6^, 2.52 × 10^−3^ for GL2, TV, and GL1, resp.) between methods with and without **m**. The QSM reconstruction image quality was improved (Table 1 score for group 1) from MGL2 to MTV (*p* = 4.98 × 10^−8^), MGL2 to MEDI (*p* = 5.44 × 10^−8^), and MTV to MEDI (*p* = 1.16 × 10^−3^).

**Figure 3:**
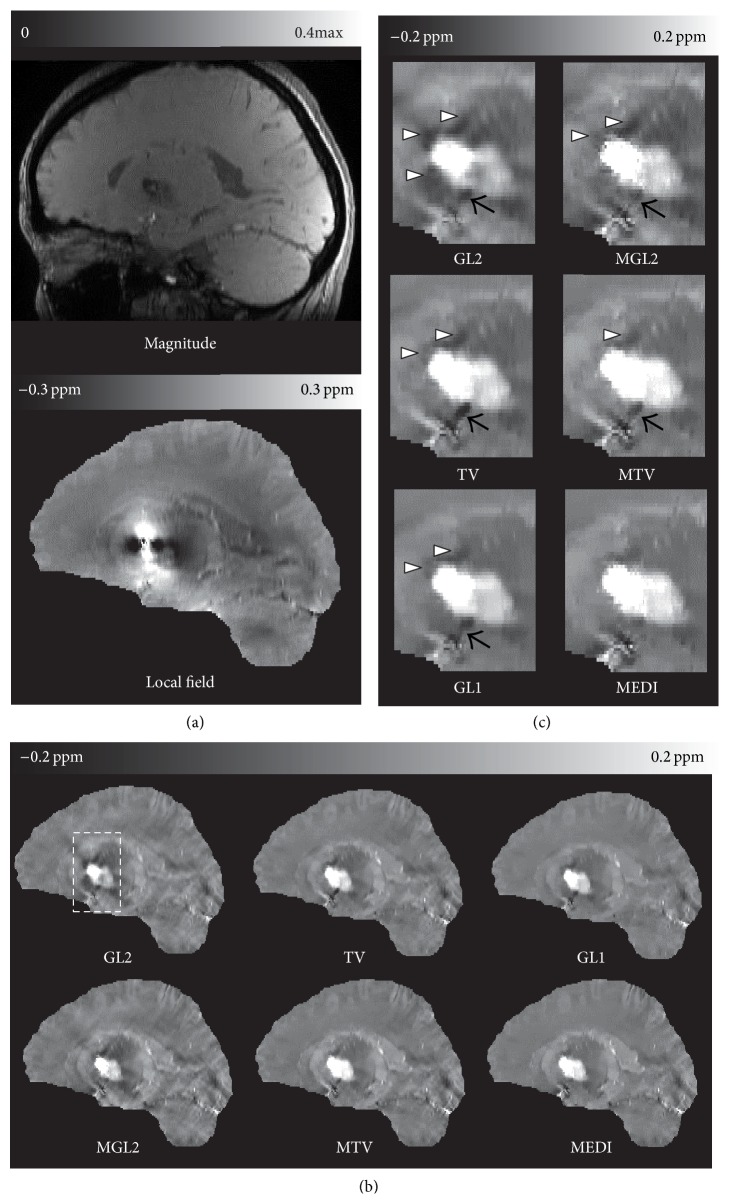
Structure regularization effects on a patient with hemorrhages in group 2. (a) magnitude, local field, and (b) reconstructed QSM images without (left) or with structure prior (right) in a sagittal section. The dashed boxes in (b) are zoomed in on (c). Similar to healthy subject images, the structure consistency **m** reduces overall streaking artifacts comparing QSM methods with and without it. The streaking artifacts were seen originating from vessels (black arrows) and the hemorrhage (hollow arrow heads) in GL2 and MGL in (c). This artifact was reduced to some extent in TV and MTV and even further in GL1 and MEDI. The averaging image quality scores of this patient were 1, 2, 2, 3.3, 3.3, and 4 for GL2, MGL2, TV, MTV, GL1, and MEDI, respectively.

The spatial prior either sharpened the image or reduced streaking artifacts (Figure 3). The differences in overall image quality (Table 1 score for group 2) were statistically significant (*p* = 4.66 × 10^−2^, 1.89 × 10^−5^, 1.80 × 10^−4^ for GL2, TV, and GL1, resp.) between reconstructions with and without structural consistency prior method when they are performed in group 2. The QSM reconstruction image quality improved from MGL2 to MTV (*p* = 9.47 × 10^−7^), MGL2 to MEDI (*p* = 3.19 × 10^−7^), and MTV to MEDI (*p* = 7.21 × 10^−4^).

The inter- and intraobserver results are shown in Table 2. These agreements ranged between good and excellent referred to the clinically relevant agreement.

## 4. Discussion

Our results of the various QSM methods indicate that QSM quality improves using a physical prior specific to the imaging situation. Investigations with a numerical Zubal lesion phantom, a Gd phantom, and 36 consecutive patients consistently corroborated this observation. These experimental results are concordant with the theoretical error analysis in QSM that the error in the reconstructed susceptibility comes from noise in the data and error in the prior [1, 19].

The ill-posedness of the dipole inversion sets up a very challenging problem for QSM. The iterative solvers employed in more accurate QSM algorithms make it difficult to understand the contributions from various terms and parameters. The systematic comparison of various methods presented in this study tends to suggest the structure prior is useful to QSM methods using the regularized minimization. This observation is established in the comparisons in Table 1. The structural matching between the magnitude image and susceptibility map by matching their gradients tends to improve the QSM image quality and accuracy as seen in the paired comparison with and without using the magnitude gradient in Figures 1 [Fig fig2]–3. It should be pointed that the new simulation with the different brain components' susceptibility has been run and the real cases were also particularly acquired with slightly different parameters in different time compared to our previous simulation and real cases in [17].

The value of the phantom experiment may be limited for assessing the performance of QSM algorithms, because the phantom was made of sphere of uniform [Gd] with perfectly identifiable edges. According to the above observation on QSM algorithms, structural information as defined by tissue contrast is a very important determinant for QSM performance. The tissue contrast of a human brain is much more subtle and complex than that of the phantom made of spheres. So what is learned from the phantom experiment is only the accuracies that various QSM methods can achieve under an ideal contrast situation. It should be pointed out that the susceptibility range of phantom experiment was much higher than that of* in vivo* human brain and the measurements were consistent with our simulation at similar susceptibility values. QSM algorithm comparison behaves similarly at a wide range of susceptibility values, which may be explainable with dimension analysis. When susceptibility is scaled and the regularization parameter is also scaled correspondingly (unchanged for L2 and scaled by the same factor for L1 and TV), the performances of the QSM algorithms remain the same.

It is also observed that GL1 is better than GL2 for structural matching (Figures 1 [Fig fig2]–3), which is consistent with previous observations that the sparsity in the prior term can be achieved more effectively with the L1 norm than the L2 norm [28, 34]. A recent publication also reached a similar conclusion that the L1 norm promoting the sparsity of spatial gradient is a better fit for QSM problem than the L2 norm [23, 35]. GL1 and TV based methods both can be categorized into L1 norm QSM algorithm. It was also noted that GL1 is better than TV methods (Figures 1 [Fig fig2]–3). This may be due to the fact that the cost of a particular edge in the objective function is the same regardless of its orientation in the TV norm, whereas the cost in the GL1 norm is dependent on the edge orientation. When there is no strong streaking artifacts as in Figure 2, the difference between GL1 and TV or between MEDI and MTV is small. When there is a large amount of streaking artifacts as in the hemorrhage case in Figure 3, the edge orientation sensitivity in GL1 and MEDI becomes obviously advantageous over the TV and MTV in suppressing the streaking artifacts.

The results by different solvers used in this paper, that is, LDFP and CG, show the consistency improvement of using structure prior. There are other solvers available including interior point methods or split Bregman method [36]. The reconstructed QSM image may be affected by the solver used in a given algorithm. However, in order to obtain reasonable answers to the concerning question on solver effects, the error propagation and accumulation in each solver may require detailed quantitative and analytic evaluation; other well-known solvers may need to be implemented for all major QSM algorithms and included for comparison. However, this important work is beyond the scope of this paper.

Some folds in cortex in Figures 2 and 3 seem to be smoothed in MEDI; this may be interpreted as that the gradient consistency between magnitude image and susceptibility map used in current MEDI is still imperfect. Indeed, recent studies have suggested that the structure prior in current MEDI may be improved with more sophisticated identification and matching of structures for more accurate QSM, such as incorporating edges derived from the local field map [37, 38]. It is also theoretically proven that a comprehensive detection of all the edges in the true susceptibility distribution will reduce the error in the reconstructed QSM [19]. However, the perfect prior as footstone of accurate regularization QSM method is still an ongoing research.

Based on its intrinsic characters, QSMs have found its power in differentiating diamagnetic calcification from paramagnetic materials [7, 39], quantifying the deoxyhemoglobin concentration [9, 12], depicting the deep brain structure [40], quantifying contrast agent concentration [41], characterizing white matter fiber tracks, and so forth [1–4, 42]. The potential clinical usefulness of QSM also has been shown in multiple sclerosis [6, 43], lupus erythematosus [44], Alzheimer's disease [45], Parkinson's disease [46], multifocal leukoencephalopathy [47], cerebral perfusion [41], hemorrhage [9, 48], function MRI [12, 42] in brain, and some applications in aorta, breast, extremity, kidney, and so forth [1–4, 42, 49]. Improving accuracy of QSM will profit these approaches.

## 5. Conclusions

In summary, the structure prior with an effective match can improve the accuracy of QSM. Among compared methods, the MEDI method appears to be the most robust for quantitative susceptibility mapping. The more accurate priors or more physically meaningful priors should be studied in the future.

## Figures and Tables

**Table 1:** Comparison of various regularization schemes for QSM.

Methods	Equation	Image quality	
		Group 1	Group 2
GL2	$\chi^{*}=\underset{\chi }{\arg\min}\left[E+\alpha {{\left\|G\chi \right\|}_{2}}^{2}\right]$	1.72 ± 0.43	1.37 ± 0.47
TV	$\chi^{*}=\underset{\chi }{\arg\min}\left[E+\alpha TV\left(\chi \right)\right]$	2.53 ± 0.40	2.13 ± 0.44
GL1	$\chi^{*}=\underset{\chi }{\arg\min}\left[E+\alpha {\left\|G\chi \right\|}_{1}\right]$	3.44 ± 0.47	2.96 ± 0.59
MGL2	$\chi^{*}=\underset{\chi }{\arg\min}\left[E+\alpha {{\left\|mG\chi \right\|}_{2}}^{2}\right]$	2.01 ± 0.08	1.70 ± 0.43
MTV	$\chi^{*}=\underset{\chi }{\arg\min}\left[E+\alpha TV\left(m\chi \right)\right]$	3.39 ± 0.46	3.15 ± 0.56
MEDI	$\chi^{*}=\underset{\chi }{\arg\min}\left[E+\alpha {\left\|mG\chi \right\|}_{1}\right]$	4.01 ± 0.53	3.74 ± 0.60

*E* = ‖**w** **z**‖_2_^2^, with **z** = **d** ⊗ **χ** − **δ**_*b*_.

$TV\left(m\chi \right)=\sum_{r}^{}m\sqrt{{\left(\partial \chi /\partial x\right)}^{2}+{\left(\partial \chi /\partial y\right)}^{2}+{\left(\partial \chi /\partial z\right)}^{2}}.$

**Table 2:** Inter- and intraobserver variability.

Method	Interobserver variability^a^	Intraobserver variability
GL2	0.85 (0.76, 0.92)	0.89 (0.79, 0.94)
TV	0.64 (0.47, 0.78)	0.79 (0.62, 0.88)
GL1	0.73 (0.59, 0.84)	0.86 (0.74, 0.92)
MGL2	0.74 (0.60, 0.85)	0.84 (0.71, 0.91)
MTV	0.66 (0.49, 0.79)	0.76 (0.58, 0.87)
MEDI	0.65 (0.49, 0.79)	0.84 (0.70, 0.91)

Data are intraclass correlation coefficients, with 95% confidence intervals in parentheses.

^a^Data are from the first reading of observer 1.

## Appendix

For ease of reproduction of all algorithms in this study, the pseudocode of each implementation is specified in this appendix.

### A. Implementation of MGL2

function MGL2(*α*)
*W* ← diag(**w**)
*M* ← diag(**m**)
*A* ← 2real[(*WF* ^−1^ *DF*)^*H*^ *WF* ^−1^ *DF*] + *α*(*MG*)^*H*^ *MG*
*b* ← 2real[(*WF* ^−1^ *DF*)^*H*^ *Wδ*_*b*_]
*χ* ← ConjugateGradient  (*A*, *b*)
return *χ*
**end function**

### B. Implementation of MEDI&MTV

function MEDI&MTV(*α*, Ψ)
*χ* ^(0)^ ← 0
*n* ← 0
switch method
  case MTV
    (B.1) $$\begin{array}{c} \Psi x=TV\left(\;x\;\right) \end{array}$$
  case MEDI
    (B.2) $$\begin{array}{c} \Psi x=G\left(\;x\;\right) \end{array}$$
end switch
*W* ← diag(**w**)
*M* ← diag(**m**)
*μ* ← 1*e* − 8
$V\left(x\right)=diag\left(1/\sqrt{x^{*}x+\mu }\right)$
while ‖*p* ^(*n*)^‖_2_/‖*χ* ^(*n*)^‖_2_ ≥ 1*e* − 2  or  *n* ≤ 10
  *A* ← 2real[(*WF* ^−1^ *DF*)^*H*^ *WF* ^−1^ *DF*] + *α*(*M*Ψ)^*H*^ *V*(*M*Ψ*χ* ^(*n*)^)*M*Ψ
  *b* ← 2real[(*WF* ^−1^ *DF*)^*H*^ *Wδ*_*b*_] − *Aχ* ^(*n*)^
  *p* ^(*n*)^← ConjugateGradient (*A*,* b*)
  *χ* ^(*n*+1)^ ← *χ* ^(*n*)^ + *p* ^(*n*)^
  *n* ← *n* + 1
end while
return  *χ* ^(*n*)^
end function
